# Disease staging according to international scoring system in newly diagnosed patients with multiple myeloma

**DOI:** 10.12669/pjms.35.1.173

**Published:** 2019

**Authors:** Saira Parveen Shaikh, Syed Muhammad Irfan, Sadia Sultan Sheikh

**Affiliations:** 1 Dr. Saira Parveen Shaikh, Department of Hematology, Liaquat National Hospital, Karachi, Pakistan; 2 Dr. Syed Muhammad Irfan Sheikh, Department of Hematology, Liaquat National Hospital, Karachi, Pakistan; 3 Dr. Sadia Sultan Sheikh, Department of Hematology, Liaquat National Hospital, Karachi, Pakistan

**Keywords:** Newly diagnosed multiple myeloma, international scoring system, disease staging

## Abstract

**Objective:**

To determine the frequency of disease staging according to international scoring system in patients who are newly diagnosed with Multiple Myeloma (MM) at a tertiary care hospital at Karachi.

**Methods:**

This single center, non probability consecutive, cross sectional study was conducted from Nov 11, 2015 to May 11, 2016. After taking informed written consent, detailed history was taken and serum β2 microglobulin and albumin levels were checked to assess the study outcome variable i.e. stage of MM. All the collected information was entered in the prescribed performa.

**Results:**

Eighty newly diagnosed patients with multiple myeloma as per inclusion criteria were included. Sixty seven (83.75%) were male and 13(16.25) were females, with mean age of 58.35+10.077 years. Twenty seven patients (33.75%) were found to have stage-I disease, in 23 (28.75%) stage-II and stage-III in 30 (37.5%).

**Conclusion:**

Multiple myeloma is relatively common in 5^th^ decade, with male predominance. International Staging System have great potential for characterizing and stratifying multiple myeloma and revealed a predominance of advanced stage III disease in our setting.

## INTRODUCTION

Multiple myeloma (MM) is a disorder of plasma-cell branded by existence of clonal growth of malignant plasma cells in the monoclonal protein in the blood, bone marrow, urine and related organs which are not functioning properly.^1^,^2^ Quarter of patients diagnosed with (MM) disease die within three years and other patients maintaining durable disease control for 10 years.^3^,^4^

The diagnosis of multiple myeloma usually requires 10% or more clonal plasma cells on bone marrow examination and proof of associated end-organ damage.^5^ It is responsible for about 1% of all neoplastic disease and percentage of 13% for hematological cancers. The average age at diagnosis is around 70 years. The percentage of patients with age below 65 years is 37%, 26% patients are within the ages of 65 and 74 years and 37% are more than 75 years of age.^1^

The patients having renal impairment, anemia, hypercalcemia and bone disease, the patients who are suffering from symptomatic myeloma need treatment, whereas patients with asymptomatic myeloma only require a regular followup.^6^ MM is a diversified disease group with respect to clinical course, reply to prognosis and therapeutic interventions. Even though multiple prognostic factors have been recognized but still there is need of best prognostic indicators

For the proper staging systems,^7^ in many studies it is proven that low serum albumin level, high lactate dehydrogenase levels, high β_2_microglobulin levels as well as host factors such as advance age and poorer performance status adversely affects the outcome. Two intrinsic characteristics i.e. cytogenetics and plasma proliferative activity can also play a vital role in the disease outcome.^7^

International Staging System (ISS) a scoring system published in 2005 by the International Myeloma working group, disease is stratified in three groups i.e;^8^

**STAGE 1:**β_2_microglobulin (β2M) levels were < 3.5 mg/L and albumin≥ 3.5 g/dl.
**STAGE 2:**β_2_microglobulin<3.5 mg/L and albumin <3.5 g/dL OR β_2_microglobulin levels between 3.5–5.5 mg/L irrespective of the serum albumin.
**STAGE 3:**β_2_microglobulin ≥ 5.5 mg/L.

The objective of our study is to determine ISS scoring in our inhabitants as ISS staging system is a widely applicable prognostic staging system for multiple myeloma patients. ISS is an inexpensive, easy and less complex than previously defined complex systems for assessing the disease status of myeloma patients. By its application early in the course of disease, we can advise the patient for disease prognosis and urgency for treatment and more importantly it will predict the median survival in our patients.

## METHODS

This single center, non probability consecutive, cross sectional study was conducted from Nov 11, 2015 to May 11, 2016. Study population in the inclusion criteria was either gender with 40 to 80 years of age, who were diagnosed cases of multiple myeloma, in the outpatient clinics and inpatients attending department of hematology at Liaquat National Hospital Karachi. Patient’s name, age, gender, address, medical record number and contact number was recorded. β2microglobulin levels was determined by Photometric methodology through Hitachi 912 instrument and their albumin levels by Chemiluminescence method through Immulite 2000 instrument. Results were recorded on a performa by researcher. The final study outcome i.e; stages of multiple myeloma were recorded on approved performa. Patients with monoclonal gammopathy of undetermined significance (based on Bone marrow biopsy <10% plasma cells), plasma cell leukemia and smoldering myeloma/ relapsed myeloma were excluded. The protocol of this study was approved by Ethical Board Review Committee at Liaquat National Hospital Karachi, Pakistan and College of Physicians and Surgeons, Pakistan. Principal investigator recorded all demography and clinical history, before enrolment informed written consent was taken to avoid confounding variables exclusion criteria was followed.

### Statistical Analysis

For analyzing the data SPSS version 22 was used. For calculation of quantitative variables like age, frequency and percentage mean and standard deviation was computed and for qualitative variables i.e. gender and stages of multiple myeloma. Satisfactory data was taken in correspondence to gender and age. Chi-square test was applied for post satisfaction; p-value ≤0.05 was taken as significant

## RESULTS

**Graph.1:**
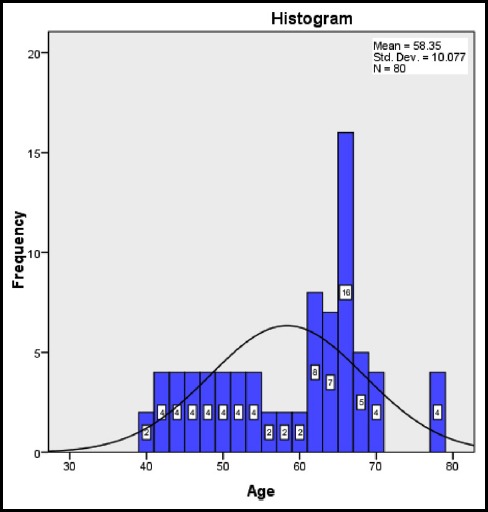
Frequency Distribution of Age.

Total 80 participants with newly diagnosed multiple myeloma as per inclusion criteria were included. Sixty seven patients (83.75%) were male and 13(16.25) were females (Table-I), with mean age of 58.35+10.077 years as shown in Table-II. The frequency distribution of age is given in Graph.1. The mean β2microglobulin level was 4.3750+1.61108 gm/dl and the mean serum albumin level was 3.3613+0.59545gm/dl.

**Table-I:** Frequency distribution of gender and stages of Multiple Myeloma.

Variable	Number (n)	Percentage (%)
***Gender***		
Male	67	83.75
Female	13	16.25
**Total**	80	100
***Stage of disease***		
Stage-I	27	33.75
Stage-II	23	28.75
Stage-III	30	37.5
**Total**	80	100

**Table-II:** Descriptive statistics of age, B_2_Microalbumin level and Serum Albumin level.

Statistics	Age (Years)	B_2_Microalbumin level	Serum Albumin level
Minimum	40	2.00	1.80
Maximum	78	7.10	4.00
Mean	58.35	4.3750	3.3613
Std. Deviation	10.077	1.61108	0.59545

In our study 27 patients (33.75%) were found to have stage-I disease, in 23 patients (28.75%) were stage-II and stage-III was seen in 30 patients (37.5%), as shown in Table-I. Stage-I was common in 40 to 60 years age group, while stage-II and stage-III were common in age group of 61 to 80 years. All stages were predominant in male gender. Stage-I disease is common in age group of 40-60 Years while stage-II and stage-III is common in age group of 61-80 years (Table-III). All stages of disease were predominant in male gender (Table-IV).

**Table-III:** Stage of disease according to age (n=80).

Age	Stage of disease			Total	P-value

	Stage-I	Stage-II	Stage-III		
40-60 Years	27(33.75%)	9(11.25%)	0(0%)	36(45%)	0.061
61-80 Years	0(0%)	14(17.5%)	30(37.5%)	44(55%)	

Total	27(33.75%)	23(28.75%)	30(37.5%)	80(100%)

**Table-IV:** Stage of disease according to gender (n=80).

Gender	Stage of disease			Total	P-value

	Stage-I	Stage-II	Stage-III		
Male	27(33.75%)	23(28.75%)	17(21.25%)	(83.75%)	0.000
Female	0(0%)	(%)	13(16.25%)	(16.25%)	

Total	27(33.75%)	23(28.75%)	30(37.5%)	80(100%)

## DISCUSSION

Multiple myeloma (MM) is characterized by heterogeneity in the clinical course and risk stratification is vital for prediction of prognosis. International staging system is the most valuable system for Multiple myeloma in the past ten years. This system predicts survival of newly diagnosed MM patients by using two routine and inexpensive pieces of laboratory data and separated patients into three stages with a distinct prognosis.^9^ Although ISS system was wildly used in Chinese myeloma patients in the past decade, the original analysis of ISS system from Greipp et al did not include Chinese patients data. Besides these, the survival of Multiple myeloma has dramatically altered by the introduction of new agents, and now a days the majority of patients received new agent based conduct in the first-line therapy.

ISS is an important prognostic mean to determine tumor burden and patient’s status. ISS is not accountable for natural factors which play vital role in the evolution of disease and confrontation to treatment. The CA not only portrays the forecast of patients having myeloma but also affect strategies of management and clinical performances.^10^ Survival of myeloma patients is highly affected by serum LDH even when they belong to low or intermediate subgroup of ISS.11 Hence it is proved that the merging of LDH and calcium levels into international staging system to make RISS system highly appropriate. Furthermore it should be kept under consideration that serum β2m levels may be raised in numerous benign conditions like as liver disease, chronic inflammation, renal dysfunction and few acute viral diseases apart from being predictive in lymphoproliferative malignancies, especially Multiple myeloma.11 MM is considered to be incurable disease and almost all suffering from this disease decline and ultimately submit to refractory disease. The degree of disease at deterioration, the response and type to earlier therapy as well as the time of deteriorated impact on prognosis of such patients. In this study 4.3750+1.61108 gm/dl was set as mean value of B2 microglobulin and 3.3613+0.59545 gm/dl was set as mean value of serum albumin. Kumar et al examined the results of 286 with relapsed multiple myeloma, who were hard to manage bortezoimb and were fallen back, refractory to or unentitled to receive, an IMid grounded on international staging system stage at the time of enrolment (T0) in the study, international staging system was predictive for OS following TO having median survivals of 12, 8, 4 months for international staging system and 1, 2 and 3 respectively.^12^

There is little data on the applicability of ISS in bortezomib-based treatment in the first-line therapy in literature. From previous studies we can indicate that MM patients can achieve deeper response by the use of novel agents, improved PFS and OS.^13^ A meta-analysis performed by Zou et al. showed the accumulation of bortezomib to first-line therapy did significantly prolong OS compared with conventional therapy alone.^14^ Some studies have showed that bortezomib-based regimens can improve result of patients with t(4;14), removal of chromosome 13, and removal of 17p, respectively.^15^

The ISS system was used as an independent prognostic system in the past, but it was unable to reflect the cytogenetic abnormalities of MM. Some new prognostic factors were increasingly found, such as fluorescent in situ hybridization (FISH), karyotype, and serum-free light chain.^16^,^17^ These new prognostic factors can overcome this deficiency and provide cytogenetic or molecular genetics-based risk classification for MM patients. Many efforts have been made, such as proposing a new stage system by combination of ISS with FISH.^18^ A recent study from IMWG combined international Staging System, calcium and LDH data to describe Revised International Staging System (R-ISS) by following three risk categories: R-ISS I including ISS stage I, no high-risk CA [del(17p) and/or t(4;14) and/or t(14;16)], and normal LDH level; R-ISS III including ISS stage III and high-risk CA or high LDH level; and R-ISS II including all the other combinations. The data of R-ISS were enrolled on 11 clinical trials from 2005 to 2013. All patients received new drugs based chemotherapy as up-front treatment. The five years OS rate in R-ISS I, II, and III was 82%, 62%, and 40%, respectively. The R-ISS system can predict prognosis on OS in patients who did receive proteasome inhibitor based treatment, while in our study the ISS system cannot clearly distribute the OS of MM patients in ISS stages I and II. One interpretation might be that, compared with R-ISS system, ISS system may wrongly allocate a certain group of patients with poor prognosis in lower ISS stage.^19^

As far as staging is concerned, in our study 27 patients (33.75%) were found to have stage-I disease, in 23 patients (28.75%) in stage-II and stage-III was seen in 30 patients (37.5%) as compared to one of the study conducted in 2012 revealed the percentage of patients according to ISS as stage 1 were 29%, stage 2 were 38% and stage 3 were 33%.^20^ According to WHO (World health organization) Classification, the survival rate of stage one is 62 months while survival rate of stage 2 is 44 months and for stage 3 it is 29 months.^21^

Stage-I was common in 40 to 60 years age group, while stage-II and stage-III were common in age group of 61 to 80 years. All stages were predominant in male gender. Earlier, retrospective study of 1038 patients also presented that the median OS augmented from 4.6 years 6.1 years for years 2001-2005 cohort v/s 2006-2010 cohort (p=0.02). The development in results was related to usage of one more new agents like bortezmib, thaidomide and lenaliomide.^3^ In USA the drugs like (Ixazomib, aratumumab, panobinostat, elotuzumab) were permitted for the treatment of Multiple myeloma in 2105. Hence we expect that the influence of combination of these agents will be realized over time.

There are many restrictive conditions for these new variables. For example, no consensus in standard classifications, not being easily available, and being too expensive. Their applications were limited by these passive factors. Thus, although novel prognostic factors such as FISH, karyotype and serum-free light chain are important in MM risk formation, the prognostic value of traditional serum markers still deserves attention. It can be an important component of new staging system in the future. Re-evaluating the prognostic value of ISS system now is beneficial for the future research for a new staging system. More ever comprehensive studies are required with larger sample size.

### Limitations of the study

It was small size of the sample. Eighty patients were taken into consideration and were enrolled in this study due to unfinished data and there were also different Hematologists involved with missing case notes or exclusion criteria.

## CONCLUSION

Multiple myeloma is relatively common in 5^th^ decade, with male predominance. International Staging System have great potential for characterizing and stratifying MM and revealed a predominance of advanced stage III disease in our setting.

## RECOMMENDATION

The international staging system is modest and influential prognostic system and here we recommend use of this system clinically to stratify patients with NDMM with respect to the relative risk to their existence.

### Authors` Contribution

**SPS:** Collected the data and analyzed it.

**SMIS:** Reviewed the manuscript and approved it.

**SSS:** Prepared the whole manuscript.
